# Macroscopic evidence for Abrikosov-type magnetic vortexes in MnSi *A*-phase

**DOI:** 10.1038/srep22101

**Published:** 2016-02-26

**Authors:** I. I. Lobanova, V. V. Glushkov, N. E. Sluchanko, S. V. Demishev

**Affiliations:** 1 Moscow Institute of Physics and Technology, 9 Institutsky lane, Dolgoprudny 141700 Moscow Region, Russia; 2 Prokhorov General Physics Institute of RAS, 38, Vavilov str. 119991 Moscow, Russia

## Abstract

Intrinsic phase coherence between individual topologically stable knots in spin arrangement – skyrmions – is known to induce the crystalline-like structure in the *A*-phase of non-centrosymmetric MnSi with chiral spin-orbit interaction. Here we report the experimental evidence for two types of the skyrmion lattice (SL) inside the *A*-phase of MnSi, which are distinguished by different coupling to the anisotropic magnetic interactions. The transition between these SLs is shown to induce a change in magnetic scattering between isotropic MR discovered in the area inside the *A*-phase (the *A*-phase core) and anisotropic MR found on the border of the *A*-phase. We argue that the SL in the *A*-phase core corresponds to the dense skyrmion state built from individual skyrmions in a way similar to Abrikosov-type magnetic vortexes.

Identifying of universal features in cooperative phenomena induced in quantized vortex matter of different origins (^3^He superfluid, triplet superconductors and chiral magnets)^1^,^2^,^3^ stays a real challenge for physical community. More than 40 years ago Dudko *et al*. discovered an analogy between the magnetization of type-II superconducting mixed state and antiferromagnet close to the magnetic field induced transition between antiferromagnetic and paramagnetic phases^4^,^5^. Taking FeCO_3_ as an experimental example, these authors were the first who calculated vortex-like state in the plane perpendicular to external magnetic field appeared due to the negative surface (domain wall) energy as a result of the specific character of exchange interactions^5^. Later on, Bogdanov and Yablonskii pointed out that such a “mixed state” of various magnets may exist due to symmetry factors staying thermodynamically stable in the systems without inversion center^6^,^7^. At that time this mixed state of magnets was definitely considered as a very similar or even perfect analogue of Abrikosov vortexes lattice^4^,^5^,^6^,^7^.

Nowadays magnetic vortexes in non-centrosymmetric magnets are generally considered as skyrmions, which are topologically stable knots in the vector field describing magnetization distribution inside the sample^1^. Historically, manganese monosilicide, MnSi, played an outstanding role in establishing of the skyrmion physics in magnets. Magnetic order, which develops in this material for *T* < *T*_*c*_ ~ 29 K, corresponds to a spiral state with the long pitch ~18 nm exceeding essentially the size of the unit cell^8^. This type of magnetic structure is formed under the competition of the strong ferromagnetic exchange *J* ~ 2.5 meV and weak Dzyaloshinskii-Moriya (DM) interaction *D* ~ 0.4 meV^9^. The skyrmion lattice (SL) state is believed to be hosted by a small pocket on the *B-T* magnetic phase diagram (*A*-phase), existing in the vicinity of *T*_*c*_ at moderate magnetic fields^10^,^11^,^12^. The SL interpretation of the *A*-phase was first deduced both from neutron scattering experiments^11^ and observation of the topological Hall effect^12^. These findings were recently confirmed by direct observation of the SL in Lorentz TEM, firstly in thin layers of the MnSi obtained from the bulk crystals^13^ and later on in the MnSi epitaxial films^14^. In theory, the skyrmion phase in MnSi may be treated as compromise between the DM and Zeeman energies. Qualitatively, the skyrmion keeps the spiral configuration of spins inside and ferromagnetic one outside, which results in gaining in both DM and magnetic field energies^15^.

However, the understanding of the *A*-phase within the SL approach raises several controversial issues, which have not been resolved up to now. First of all, the existing theory proves the skyrmion-based states to be stable only in two-dimensional case, and additional mechanisms should be involved to stabilize the SL state in bulk MnSi^11^,^15^,^16^,^17^. The surprising disappearance of the *A*-phase recently observed in MnSi epilayers for the out-of-plane external magnetic field leads authors of ref. 17 to a conclusion that the only thermodynamically stables phases on the *B-T* phase diagram are the conical (C) phase, ferromagnetic (or spin-polarized, SP) phase and paramagnetic (P) phase and the *A*-phase is completely metastable in 3D case. These results were disputed in^18^, where a comprehensive Lorentz TEM study of the MnSi thin plates of various thicknesses prepared from the single crystals showed the presence of the SL states regardless the crystal orientation with respect to the field. At the same time both refs 17 and 18 agree on the valuable contribution of the magnetic anisotropy and thermal fluctuation effects for the skyrmion stability.

Secondly, Grigoriev *et al*. pointed out recently that the term “skyrmion lattice” currently implies two mutually exclusive physical realizations of the *A*-phase in the MnSi^19^. The first one is very close to the initial concept of Abrikosov-type magnetic vortexes. According to the basic study^16^ the skyrmions are real quasi-particles, which may build hexagonal SL. The period of the SL is not necessary related to the spiral pitch, although it should be of the same order of magnitude^15^. In this approach the SL may melt into smaller skyrmion-based clusters and skyrmion-like magnetic fluctuations may be expected above *T*_*c*_^20^. The second concept originates from the *A*-phase model based on the triple-***k*** spin structure stabilized by Gaussian thermal fluctuations under magnetic field^11^. The latter ansatz accounts for skyrmion-like topology, which includes protected knots and windings in the *A*-phase magnetic structure^11^,^12^. Therefore this type of SL is nothing but a complicated magnetic phase, which could not decay into individual skyrmions. The search for the SL melting effects performed in^19^ provided negative result, leading to the conclusion that quasi-particle skyrmion scenario of the *A*-phase is not correct.

The analysis of the current problematics of the SL state in the bulk MnSi allows formulating two problems, which should be solved experimentally. 1. Does the *A*-phase be really metastable or demonstrate any features of metastability at least? 2. How is the SL state coupled to the crystalline lattice? Following Grigoriev conjecture^19^, it is natural to expect that the triple-***k*** spin structure is somehow coupled to the lattice via anisotropic magnetic interactions, which may result in the noticeable anisotropy of the *A*-phase boundary as reported previously in numerous experiments. From the other hand, the SL based on the Abrikosov-type magnetic vortexes should be less dependent from magnetic anisotropy and thus may by decoupled from crystallographic directions. Additionally, the results of ref. 17 require a comprehensive study of the effects to be appeared under various orientations of the external magnetic fields with respect to the crystal axes. Finally, as long as any model of the SL state in MnSi results in six-fold symmetry of the diffraction pattern for magnetic scattering, it is desirable to use alternative experimental methods for solving the aforementioned problems.

In the present work, we report the result of the experiment consisting in an investigation of the angular dependences of the magnetoresistance (MR) inside and near the *A*-phase *B-T* domain in the bulk MnSi single crystals. The magnetic scattering of charge carriers is known to dominate in MnSi under negligible Boltzmann contribution to MR^21^, so that this physical parameter happens to be very sensitive to the magnetic structure. Therefore studying of the MR anisotropy may bring additional information, which is hardly accessible in direct structural studies. It is worth noting that previous studies of MR^10^,^21^,^22^ discussed the data having no relevance to specific crystallographic direction or corresponding only to a limited set of magnetic fields orientations. Additionally, the majority of MR experiments were performed outside the *A*-phase area in the magnetic phase diagram of MnSi^10^,^21^,^22^. Our precise measurements of MR carried out just in the diapason of *B-T* parameters, where the topologically non-trivial magnetic structure may exist, and the subsequent analysis of the MR data allow concluding that the *A*-phase in the bulk MnSi (a) is not metastable; (b) includes the region, where the SL state is decoupled from the crystalline magnetic anisotropy. We argue that this region on the magnetic phase diagram most likely represents the magnetic vortex state constructed of individual skyrmions. The obtained results may provide a new look on the problems of skyrmion stability in the 3D case and melting of the SL.

We start from the analysis of the field dependences of the MR for magnetic fields applied along the main crystallographic directions [001], [0-11] and [1-11] (Fig. 1a), which can be used to determine the magnetic phase diagram for the studied MnSi sample^10^. The typical ρ(*B*,*T* = const) data are presented in Fig. 1b (T = 27.7 K). The most pronounced kinks correspond to magnetic transitions between cone (C) and spin-polarized (SP) phases (arrows 1 in Fig. 1b) and to the phase boundaries of the *A*-phase (arrows 2a,b in Fig. 1b). The transition between helix (H) and C phases (arrows 3 in Fig. 1b) does not influence significantly on the ρ(*B*,*T* = const) curves but becomes visible as a particular feature (maximum or minimum) of the derivative ∂ρ/∂*B* (inset in Fig. 1b). The analysis of the ρ(*B*,*T*) field dependences allowed obtaining the magnetic phase diagram (Fig. 2). It is obvious that the largest pocket of the *A*-phase corresponds to the case **B**|| [001]. Comparison with the known magnetic phase diagrams for MnSi extracted from the magnetotransport and magnetization measurements^10^,^11^,^12^,^23^ demonstrates a reasonably good agreement of the present results (Fig. 2) with that ones reported previously.

According to suppositions made in ref. 17, not only the kink on the ρ(*B*) curves but also magnetic hysteresis is an essential fingerprint of the “metastable” *A*-phase on the magnetic phase diagram. This statement is based on the pioneering results^10^, where strong MR hysteresis was found for the up and down sweeps of magnetic field. As the metastable nature of the *A*-phase excludes it from the set of “true” magnetic phases^17^ the hysteresis effects may be attributed to the metastability. In Fig. 1b the accuracy of the temperature stabilization during field scans was ~3 mK and the ρ(*B*) data for sweeping up (solid lines) and sweeping down (dashed lines) almost coincide. The magnetic hysteresis became more pronounced only when the stabilization level was increased to ~5 mK. These observations clearly show that hysteresis features may be completely attributed to the thermal effects occuring at the *A*-phase boundaries. Indeed, from the theoretical point of view the transitions between the C phase and *A*-phase should be the first order ones^15^. Therefore, the *A*-phase in MnSi has well-defined phase boundaries similar to another phases on the *B-T* diagram and hence possible hysteresis effects^10^,^17^ are nothing but experimental artefacts caused by discrepancy between the measured and real sample temperature.

A remarkable feature of the ρ(*B*) dependences in Fig. 1b is a coincidence of the resistivity values for three main crystallographic directions in the interval around 0.2 T. It corresponds to the area on the *B-T* magnetic phase diagram, which is common for the three *A*-phase pockets in Fig. 2 (hereafter this area will be denoted as *A*-phase core). At the same time, the ρ(*B*) curves exhibit strong anisotropy outside of the specific interval (Fig. 1b).

In order to analyze the magnetic anisotropy effects inside and around the *A*-phase, the angular dependences of the resistivity ρ(φ) were investigated along different lines on the *B-T* magnetic phase diagram (dashed lines a–d in Fig. 2). Three cuts were taken at fixed temperatures *T* = 26.9 K, *T* = 27.7 K, *T* = 28.9 K under varying magnetic field (lines a–c in Fig. 2) and one cut was measured at fixed magnetic field *B* = 0.194 T under varying temperature (line d in Fig. 2). Figure 3 presents the resultant normalized ρ(φ)/ρ(0) data shifted for clarity along y axis. The isotherm *T* = 26.9 K lying outside of the *A*-phase (section a in Fig. 2) captures the sequence of the magnetic phase transitions H → C → SP. It is visible (Fig. 3a) that strongest magnetic scattering and the maximal resistivity occur for **B**||[001] (or equally for **B**||[00–1]) in all these magnetic phases (H, C and SP) although the shape of the ρ(φ) curves varies. Moreover, the direction of the maximal magnetic scattering **B**|| [001] remains the same in both SP and P phases (see Fig. 3a,d).

On the contrary, the *T* = 28.7 K and *B* = 0.194 T cuts, which cross the *A*-phase (lines c and d in Fig. 2), demonstrate qualitatively different behavior of resistivity for the region inside the *A*-phase core. In this region of the magnetic phase diagram the resistivity not only coincide for the main crystallographic directions (Fig. 1b), but becomes completely isotropic so that ρ(φ) = const for *any* angle (Fig. 3c,d). This characteristic feature, which does not depend on the section type (*T* = const or *B* = const), allows to conclude that MnSi “forgets” any magnetic anisotropy related to the crystalline lattice inside the *A*-phase core. This fact can be easily recognized within the theory of the mixed state of magnets to be analogous to a mixed state of superconductors^4^,^5^,^6^,^7^. Indeed, the Abrikosov-like magnetic vortexes are superstructural entities, which are linked to the direction of magnetic field rather than to any symmetry of crystal. Therefore the observation of the ρ(φ) = const looks reasonable for a state built on the magnetic vortexes similar to those ones in type II superconductors.

Our experimental data suggest that the flat portions on the ρ(φ) angular dependence may be used to determine the stability regions for the SL phase on the magnetic phase diagram. On the basis of this supposition it is possible to analyze transition between the *A*-phase core and surrounding magnetic phases. It is instructive to consider entering into the *A*-phase region for **B**|| [001] and **B**|| [00–1] (Fig. 3b,c). We see that for the scan which does not pass through the *A*-phase core (Fig. 3b) the regions ρ(φ) = const exists only in the angular range up to Δφ(*B*) ~ ±20^o^ from [001] or [00-1] axes (dashed lines in Fig. 3b). Outside this interval the angular dependences of the resistivity keeps the same behavior as in the C phase. Therefore it is reasonably to suppose that magnetic vortexes forming SL state are somehow linked with the selected crystallographic direction ([001] in the considered case) and may exist only when the deviation of the magnetic field direction does not exceed critical value Δφ(*B*) (Fig. 3b). It is also worth noting that angular diapason of the magnetic vortex state stability is marked by characteristic kinks on the ρ(φ) dependence. Therefore we assumed that the shape of the ρ(φ) curve the intermediate magnetic region is formed by superposition of magnetic scattering in the *A*-phase inside interval Δφ(*B*) and in the cone phase outside of the Δφ(*B*) (see curves marked as A + C in Fig. 3b) Apparently, Δφ(*B*) → 0 on the phase boundaries between the *A*-phase and cone phase for **B**|| [001] or **B**|| [00–1] (Fig. 3b). To summarize, the total angular dependences of the MR ρ(φ) for the considered scan (Fig. 3b), where transitions into *A*-phase are observed, result from contributions of both the C phase and the *A*-phase pinned around the **B**|| [001] (or **B**|| [00-1]) direction.

The intermediate ρ(φ) behavior inside the *A*-phase, which precedes the onset of the ρ(φ) = const in the *A*-phase core, is also evident from Fig. 3c (see the data for B = 0.156 T). However in the latter case the angular variation of the resistivity in the *A*-phase becomes more complicated due to close phase boundaries between the C phase and *A*-phases for **B**||[001] and **B**|| [1–10] (see also Fig. 2).

When considering the *B* = const route (Fig. 3d) it is possible to note that increase of temperature results in entering into the *A*-phase along **B**|| [001], which exists in small range Δφ(*T*) around [001] or [00–1] (dashed lines in Fig. 3d). Following the above consideration, the respective ρ(φ) curves (*T* = 27.5 K and *T* = 28 K in Fig. 3d) reflect the superposition of the C phase and *A*-phase segments (similar to that ones for B = 0.19–0.24 T in Fig. 3b). When temperature reaches the *A*-phase core, the resistivity does not depend on the angle (Fig. 3d). Actually, the SL pinned along **B**||[001] (or **B**||[00–1]) direction (*A* + C curves in Fig. 3d) *abruptly* transforms into the magnetic vortex state decoupled to crystallographic axes (*A*-core curves in Fig. 3d). Within the accuracy of measurements, this transformation occurs just at the point separating the *A*-phase for **B**||[001] and *A*-phase core.

The established behavior of the magnetic vortex state in MnSi begs the evident question: can these effects be completely attributed to anisotropic *T*-*B A*-phase boundaries (Fig. 2) or does this feature reflect the real difference in magnetic states between the *A*-phase core and the rest of the *A*-phase? To resolve between these alternatives, it is instructive to analyze experimental geometry **B**||[001] for the field section *B* = const crossing the *A*-phase. Indeed, if this is a trivial case of anisotropic phase boundaries, the only phase boundaries for **B**||[001] should exist up to transition into P-phase as long as SL states for **B**||[0–11] and **B**||[1–11] and hence *A*-phase core does not exist under this assumption (Fig. 2). On the other hand, if *A*-phase core is a real magnetic isotropic phase different from any other phases on the *B-T* diagram for **B**||[001] a magnetic transition should exist between the *A*-phase and *A*-phase core.

We have analyzed the temperature dependences of the resistivity ρ(*T*) in fixed fields *B* = 0.194 T (section d in Fig. 2) and *B* = 0.308 T **(B**||[001]). The scans at *B* = 0.308 T do not cross the *A*-phase for any crystallographic direction (Fig. 2). Hence the corresponding ρ(*T*) curve may be used as a reference for elucidation of the possible magnetic scattering peculiarities along the section, which passes through the *A*-phase and the *A*-phase core. The result for the difference Δρ(*T*) = ρ(0.194 T)−ρ(0.308 T) is shown in the main panel of Fig. 4. Inset in Fig. 4 represents the initial resistivity temperature dependences for 0.194 T and 0.308 T, the last one being shifted down for 5 μΩ·cm for clarity.

The Δρ(*T*) data demonstrate a stepwise anomaly at the phase boundary between the cone phase and *A*-phase, which must exist in any model of the *A*-phase. Surprisingly the kink having ~1.7 bigger amplitude develops inside the *A*-phase at the boundary between the *A*-phase and *A*-phase core. Apparently the latter feature of magnetic scattering may be present when and only when the *A*-phase core is a new isotropic magnetic phase existing for any crystallographic direction inside the anisotropic *A*-phase domain. In the vicinity of the transition between the *A*-phase and P-phase the Δρ(*T*) data become distorted due to obvious mismatch of the transition temperatures for *B* = 0.194 T and *B* = 0.308 T (see Fig. 2). However, even in this case the peculiarities of the magnetic scattering at the transition between the *A*-phase and P-phase for *B* = 0.194 T remain visible as an inflection point at Δρ(*T*) curve (Fig. 4).

To summarize, the behavior of the resistivity established in MnSi (Fig. 4) proves definitely that MR (and hence magnetic scattering) undergoes quantitative changes at the boundary between the *A*-phase and *A*-phase core in addition to the qualitative transformation of the MR angular dependences. Therefore it is possible to conclude that, in view of magnetic scattering properties, the *A*-phase core is different from either the *A*-phase or the cone phase of MnSi.

The established boundary between the *A*-phase and *A*-phase core (Fig. 4) supports the different structure of the SL states inside the *A*-phase boundaries. The SL for the *A*-phase core seems not be linked to any particular axes in the crystal, and the only selected direction remains the direction of the magnetic field. This is definitely the case of the SL constructed from individual skyrmions. In this case a direct analogy with the vortex state of a superconductor can be proven mathematically^15^ and therefore its rotational symmetry has no confinement to any crystallographic direction. At the same time, the SL state outside the *A*-phase core is strongly coupled to particular crystallographic direction, for example, to [001] (Fig. 3b). Such behavior may be expected in the triple-***k*** spin configuration based models^11^, as long as they should imply some anisotropy effects responsible for angular dependent anisotropic boundaries of the *A*-phase. The difference between SL states inside the *A*-phase may, in our opinion, naturally explain the peculiarities of magnetic scattering observed at the boundary between the *A*-phase and *A*-phase core for **B**||[001] (Fig. 4) as well as qualitative change of the MR angular dependences (Fig. 3).

The considered ansatz may also lead to several consequences, which may open a new way for the explanation of some problems in skyrmion physics mentioned above. For instance, the most common representation of experimental data ignores any detailed angular dependences taking into account only the data for magnetic field applied along main crystallographic directions (Fig. 2). In contrary, the Abrikosov-type magnetic vortex state happens to be “surrounded” by another SL state, which is metastable in some sense^11^. The link of the SL state to particular axis in the crystal is essential and cannot be ignored. Staying on the basis of the theoretical models to be available to date, the considered assumption should correspond to the *A*-phase structure with the isotropic SL state built from individual skyrmions as quasiparticles (the *A*-phase core), which is embedded inside the anisotropic triple-**k** based SL.

As long as the skyrmions are confined inside another type of the SL for the magnetic field applied along main crystallographic directions, they are unable to decay into individual quasiparticles and no melting to individual skyrmions is possible in this model. Therefore the problem pointed out by Grigoriev *et al*.^19^ gets natural solution: instead of the rivalry the two different SL phases may coexist inside the *A*-phase.

In this respect, it possible to conclude that the study of the skyrmion-based states stability in MnSi performed to date^1^,^11^,^15^,^16^,^17^,^18^ is not complete, because the thermodynamic stability of the SL was analyzed with respect to the C and SP (ferromagnetic) phases. The complete analysis, which has not been done so far, should include evaluation of the energies for the various types of dense skyrmion phases discriminated by their magnetic anisotropy. Prior to such calculations it is not possible to come to a definite conclusion concerning the stability of the 3D SL states, which consist of the individual skyrmions.

The considered alternative explanations of the obtained experimental data show that precise analysis of the magnetic structure inside the *A*-phase with the help of direct methods like neutron scattering and Lorentz microscopy could be rewarding. In any case, our experiment shows that *A*-phase cannot be treated as magnetically homogeneous, as long as its different regions exhibit different magnetic scattering and different sensitivity of the SL states to magnetic anisotropies characterizing the B20 non-centrosymmetric structure of MnSi.

Summarizing up, the performed study of the MR angular dependences in MnSi reveals the different types of the SL inside the *A*-phase to be distinguished by coupling to the anisotropy of magnetic interactions in MnSi. The MR is found to become isotropic in the area inside the *A*-phase, which is common to all crystallographic directions (*A*-phase core), where dense skyrmion state is built from individual skyrmions in analogy to Abrikosov-type magnetic vortexes. The regions outside the *A*-phase core are characterized by relatively narrow angular ranges of the *A*-phase stability, which are strongly linked to crystallographic axes. The analysis of experimental data shows that a magnetic transition between different types of the SLs could be expected inside the *A*-phase in the bulk MnSi.

## Methods

**Figure 1:**
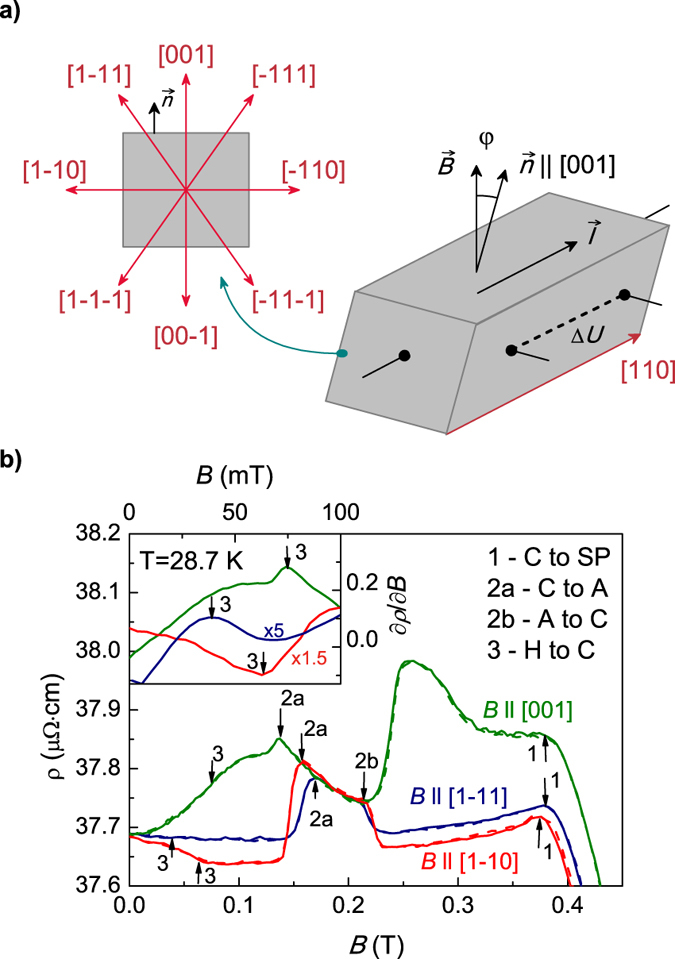
(**a**) Experimental geometry for the transverse MR anisotropy measurements. (**b**) Field dependences of the resistivity as measured at *T* = 28.7 K for magnetic fields applied along different crystallographic directions. Arrows denote magnetic transition between the conical (C) and spin-polarized (SP) phases (1), the segment of the *A*-phase stability (2**a,b**) and the phase boundary between the helix (H) and C phases (3). Inset in panel b shows ∂ρ/∂*B* data in the units of 10^−5^ Ω · cm/T.

High quality single crystals of MnSi identical to those studied earlier in^19^ were investigated. Details concerning samples preparation and characterization can be found elsewhere^19^. For MR measurements, the DC excitation current was applied parallel to [110] direction in all cases. The step motor driven installation allowed step-by-step rotating of the sample (angle step 1.8^o^) in a steady magnetic field supplied by a superconducting solenoid. The rotation of the single crystal around the [110] axis allowed changing direction of magnetic field **B** in the transverse MR experiment so that vector **B** can gradually pass in one run the main crystallographic directions [001], [1-11], [1-10], [1-1-1], and respectively [00-1], [-11-1], [-110], [-111] (Fig. 1a). The position of the crystal is identified by the angle φ between the vectors **B** and [001] (Fig. 1a). The standard four-probe technique was used to measure sample resistivity ρ(*B,T*) as a function of temperature and magnetic field, the relative accuracy of the resistivity data was about ~10^−5^ and the accuracy of temperature stabilization in the diapason 26–30 K reached ~2 mK independent on the angle φ and magnetic field magnitude up to 0.5 T.

## Additional Information

**How to cite this article**: Lobanova, I. I. *et al*. Macroscopic evidence for Abrikosov-type magnetic vortexes in MnSi *A*-phase. *Sci. Rep*. **6**, 22101; doi: 10.1038/srep22101 (2016).

## Figures and Tables

**Figure 2:**
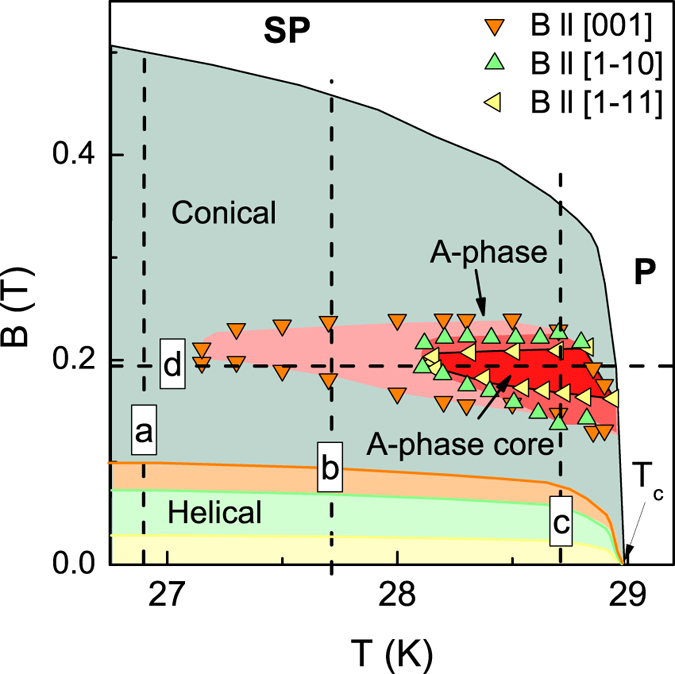
The magnetic phase diagram of MnSi reestablished from the MR measurements. Dashed lined show different cuts taken at (**a**) *T* = 26.9 K, (**b**) *T* = 27.7 K, (**c**) *T* = 28.7 K and (**d**) *B* = 0.194 T.

**Figure 3:**
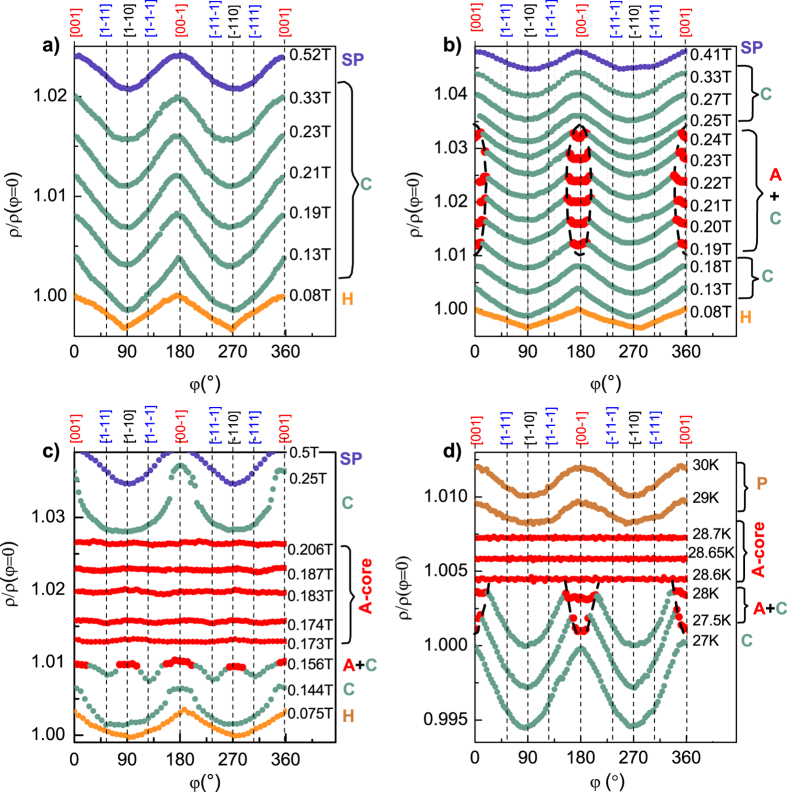
Angular dependences of the MR for the cuts of the *B-T* magnetic phase diagram taken at *T* = 26.9 K (no *A*-phase, (**a**), *T* = 27.7 K (short segments of *A*-phase, (**b**), *T* = 28.7 K (*A*-core crossing, (**c**) and *B* = 0.194 T (*A*-core crossing, d). Bold dashed lines in the panels b and d denote the segment of the *A*-phase, which appears near **B**||[001] direction. The magnetic phases corresponding to the ρ(φ) data are given at the right of each panel by the same color.

**Figure 4:**
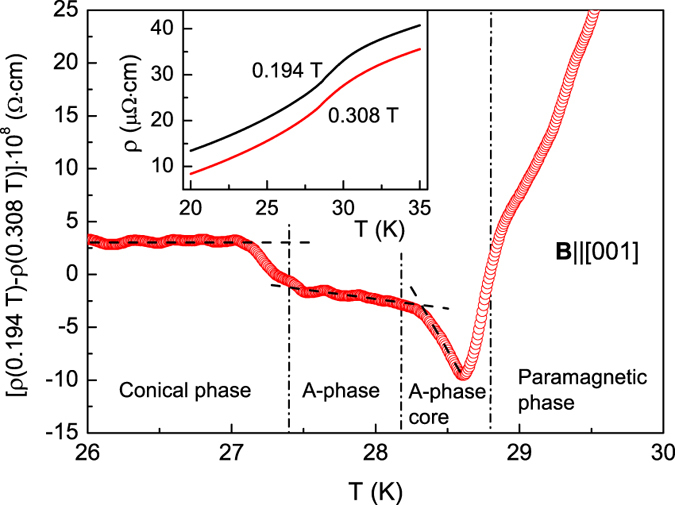
Inset: temperature dependences of the resistivity ρ for *B* = 0.194 T (cut d in Figs 2 and 3, which crosses the *A*-phase) and *B* = 0.308 T (the cut, which is outside the *A*-phase area). The ρ(*T*) curve for *B* = 0.308 T is shifted downwards by 5 μOhm·cm for clarity. Main panel: temperature dependence of the difference ρ(0.194 T)-ρ(0.308 T). Dashed-dotted lines mark the transitions between the C phase, *A*-phase, *A*-phase core and paramagnetic phase (see also Fig. 2). Dashed lines are guides for the eye denoting the different trends of MR in the magnetic phases of MnSi. In all cases, the magnetic field is applied parallel to the [001] direction.
